# Identification of a novel TIF-IA–NF-κB nucleolar stress response pathway

**DOI:** 10.1093/nar/gky455

**Published:** 2018-06-05

**Authors:** Jingyu Chen, Ian T Lobb, Pierre Morin, Sonia M Novo, James Simpson, Kathrin Kennerknecht, Alex von Kriegsheim, Emily E Batchelor, Fiona Oakley, Lesley A Stark

**Affiliations:** 1 University of Edinburgh Cancer Research Centre, Institute of Genetics and Molecular Medicine, Western General Hospital, Crewe Rd., Edinburgh EH4 2XU, UK; 2 Liver Research Group, Institute of Cellular Medicine, 4th Floor, William Leech Building, Framlington Place, Newcastle University, Newcastle Upon Tyne NE2 4HH, UK

## Abstract

p53 as an effector of nucleolar stress is well defined, but p53 independent mechanisms are largely unknown. Like p53, the NF-κB transcription factor plays a critical role in maintaining cellular homeostasis under stress. Many stresses that stimulate NF-κB also disrupt nucleoli. However, the link between nucleolar function and activation of the NF-κB pathway is as yet unknown. Here we demonstrate that artificial disruption of the PolI complex stimulates NF-κB signalling. Unlike p53 nucleolar stress response, this effect does not appear to be linked to inhibition of rDNA transcription. We show that specific stress stimuli of NF-κB induce degradation of a critical component of the PolI complex, TIF-IA. This degradation precedes activation of NF-κB and is associated with increased nucleolar size. It is mimicked by CDK4 inhibition and is dependent upon a novel pathway involving UBF/p14ARF and S44 of the protein. We show that blocking TIF-IA degradation blocks stress effects on nucleolar size and NF-κB signalling. Finally, using *ex vivo* culture, we show a strong correlation between degradation of TIF-IA and activation of NF-κB in freshly resected, human colorectal tumours exposed to the chemopreventative agent, aspirin. Together, our study provides compelling evidence for a new, TIF-IA–NF-κB nucleolar stress response pathway that has *in vivo* relevance and therapeutic implications.

## INTRODUCTION

The nucleolus is a highly dynamic, sub-nuclear organelle. In addition to its primary function as the hub of ribosome biogenesis, it acts as a critical stress sensor and coordinator of stress response (1,2). The starting point of ribosome biogenesis is transcription of ribosomal DNA (rDNA), which is mediated by the RNA polymerase I (PolI) complex. If cellular homeostasis is challenged, a variety of kinases target this complex and consequently, rDNA transcription is inhibited, the gross architecture of the nucleolus is altered, the nucleolar proteome is dramatically modified and ultimately, signals are transmitted to downstream effector pathways so that cell growth and death are altered accordingly (1–3). This chain of events is broadly termed ‘nucleolar stress’ and the most recognised and characterised effector is the MDM2-p53 axis (4–6). However, it is increasingly apparent that nucleolar stress can regulate cell phenotype in a p53 independent manner (7–10). Indeed, the mechanisms that coordinate stress effects on the PolI complex, and integrate these into individual phenotypic outcomes, remain poorly understood.

Similar to p53, the NF-κB transcription factor plays a critical role in regulating cell growth and death in response to stress (11). The most abundant form of NF-κB is a heterodimer of the p50 and RelA (p65) polypeptides which is generally bound in the cytoplasm by the inhibitor, IκBα. Upon exposure of the cell to a myriad of stresses, IκBα is degraded allowing NF-κB to translocate to the nucleus where it regulates expression of target genes (12,13). In contrast to the rapid activation observed in response to classical NF-κB stimuli, stress stimuli (including serum starvation, UV-C radiation and chemopreventative/therapeutic agents) generally induce the pathway with a much slower and delayed kinetic (14). Although a number of mechanisms have been proposed, how multiple environmental and cytotoxic stimuli induce the delayed activation of NF-κB remains unclear.

In this lab, we noted that a common response to stress stimuli of the NF-κB pathway is modulation of nucleolar architecture. In particular, an increase in the size of the organelle (14). This was of interest because a common denominator of stresses that activate NF-κB is inhibition of rDNA transcription (some of which are summarised in Supplemental Table S1) (15–17). Furthermore, proteins that have a role in stress-mediated activation of NF-κB reside within this organelle (18–21). We have previously demonstrated that post induction, RelA can accumulate in nucleoli (14,22) and others have shown modulation of NF-κB signalling by ribosomal proteins (23,24). However, to the best of our knowledge, no association between nucleolar stress and induction of NF-κB signalling has previously been reported.

Here, we investigated the relationship between stress effects on nucleoli and NF-κB signalling. We identify a novel mechanism by which stresses act on nucleoli which involves degradation of the PolI complex component, TIF-IA. We show that increased nucleolar size and activation of the NF-κB pathway are a direct downstream consequence of this degradation. Furthermore, we show that this novel TIF-IA–NF-κB nucleolar stress response pathway is triggered in a whole tissue setting and has relevance to the anti-tumour effects of aspirin.

## MATERIALS AND METHODS

### Cell lines and treatments

Human SW480, HRT18, RKO and HCT116 colon cancer cells, PNT pancreatic cells and Hela Cervical cancer cells, are available from the American Type Culture Collection (ATCC). The p53 null derivative of HCT116 (HCT116^p53-/−^) was a gift from Professor B Vogelstein (John Hopkins University School of Medicine, USA) and has previously been described (25). HRT18SR, a derivative of HRT18 cells that constitutively expresses a non-degradable IκBα, was generated in this lab and has been described (26). All cell lines were maintained at 5% CO_2_ in growth medium (Gibco) supplemented with 10% fetal calf serum (FCS) and 1% penicillin/streptomycin. Medium used was: SW480: Leibovitz's L-15: PNT, RKO, HCT116, HCT116p^53−/−^-DMEM; HRT 18, HRT18SR-RPMI with Geneticin (Gibco) selection.

All treatments were carried out in reduced serum (0.5% FCS) medium for the times and concentrations specified. Aspirin (Sigma) was prepared as previously described (26). ActinomycinD (Sigma), Cyclohexamide (Sigma), TNF (R&D Systems), Ceramide C2/C6 (Sigma), Fumonisin (VWR International), MG132 (Sigma), Lactacystin (Calbiochem), Quinacrine (Sigma), Roscovitine (Cell Signalling Technology), BafilomycinA1 (Cambridge Bioscience) and Rapamycin (Sigma) were all prepared as per manufacturer's instructions and used at the concentrations given. For UV-C treatments, cells were mock treated or exposed to UV-C under the conditions stated^.^ The CDK4 inhibitors 2-bromo-12,13-dihydro-5*H*-indolo[2,3-*a*]pyrrolo[3,4-*c*]carbazole-5,7(6*H*)-dione (CDK4i, Calbiochem) and Palbociclib (PD-0332991, Selleckchem) were solubilised in DMSO and used as indicated. The rDNA transcription inhibitor BMH-21 (12H-Benzo[g]pyrido[2,1-b]quinazoline-4-carboxamide, N-[2(dimethylamino)ethyl]-12-oxo) was kindly supplied by Prof. Marikki Laiho (Johns Hopkins University School of Medicine, USA) and Nutlin-3 by Prof. Kathryn Ball (University of Edinburgh, Edinburgh Cancer Research Centre, UK).

### Immunocytochemistry, image quantification and FUrd assays

Immunocytochemistry was performed as previously described (22). Primary antibodies were TIF-IA (BioAssayTech), Rrn3 (mouse), RelA (C-20), Nucelolin (MS-3), RPA194 (all Santa Cruz Biotechnology) and Fibrillarin (Cytoskeleton). Cells were mounted in Vectashield (Vector Laboratories) containing 1ug/ml DAPI. Images were captured using a Coolsnap HQ CCD camera (Photometrics Ltd, Tuscon, AZ, USA) Zeiss Axioplan II fluorescent microscope, 63 × Plan Neofluor objective, a 100 W Hg source (Carl Zeiss, Welwyn Garden City, UK) and Chroma 83 000 triple band pass filter set (Chroma Technology, Bellows Falls, UT, USA). Image capture was performed using scripts written for IPLab Spectrum 3.6 or iVision 3.6 in house. For each experiment, a constant exposure time was used. Image quantification was carried out using DAPI as a nuclear marker and fibrillarin as a nucleolar marker along with ScanR (Olympus), IPLab or ImageJ image analysis software as specified in figure legends. Nuclear to cytoplasmic ratios of RelA were quantified by ImageJ measuring signal intensity in a defined area of the nucleus (indicated by DAPI) and an equal area at the sub-nuclear periphery (cytoplasm). At least 100 cells, from at least five random fields of view, were quantified per experiment, for three independent experiments or as specified in the text.

For fluorouridine (FUrd) run on assays, cells were treated with 2 mM FUrd 15 min before harvest. Immunocytochemistry was then performed with an anti-BrdU antibody (Sigma). Images were captured using a ScanR high-content imager (Olympus) with a LUCPLFLN 40× objective (Olympus) and ScanR Acquisition software (Olympus). FUrd incorporation was quantified for at least 1000 cells per slide using ScanR analysis software with particle recognition algorithms.

### Plasmids, siRNA and transfections

Flag-UBF wild type, S388G and S484A mutants were kindly provided by R. Voit (German Cancer Research Centre, Heilderberg, Germany) (27). NF-κB reporter constructs (3× enhancer κB ConA (3× κB ConA-Luc), IκBα luciferase (IκBα-Luc)) and ΔκB-deleted derivatives (ΔκB ConA-Luc, ΔIκBα-Luc) were provided by RT Hay (University of Dundee, Dundee, UK) and have been described elsewhere (14). pCMV-β is commercially available (Promega). pEGFP-C1-hTIF-IA was kindly gifted by I Grummt (German Cancer Research Centre, Heilderberg, Germany). Flag-P14ARF was gifted by A Lamond (University of Dundee, Dundee, UK).

siRNA duplex oligonucleotides were synthesized by MWG and transfected into cells using lipofectamine 2000 following the manufacturer's instructions. Cells were transfected on two consecutive days then left to recover for 24–48 h prior to treatment or harvest. siRNA sequences are as follows: TIF-IA CUAUGUAGAUGGUAAGGUU; TIF-IA CUAGAAUUCCGUUCUUCUA; CDK4 AAGGCCCGUGAUCCCCACAGU UBF CCAAGAUUCUGUCCAAGAA; p14ARF AAGACCAGGUCAUGAUGAUGG; Control AGGUAGUGUAAUCGCCUUG.

### Quantitative PCR

RNA was extracted from cells using RNeasy mini kit (Qiagen) following the manufacturer's instructions. Extracted RNA was purified using RQ1 RNase-free DNase (Promega) then cDNA generated using 1st Strand cDNA synthesis kit (Roche). Taqman assays (Thermo Fisher Scientific) and a LightCycler 480 system were used to quantify transcript levels. The Comparative C_T_ Method (or ΔΔC_T_ Method) was used for calculation of relative gene expression.

### Immunoblotting, luciferase reporter, cell cycle and apoptosis assays

Immunoblotting, luciferase reporter and AnnexinV apoptosis assays were carried out as described previously (14,26). Primary antibodies used for immunoblots are as follows: TIF-IA (rabbit, 1:2000, BioAssayTech B8433); Rrn3 (Mouse, 1:500, Santa Cruz sc-390464); RPA194 (POLR1A) (H 300, Rabbit, Santa Cruz, sc-28714); RelA^S536^ (Rabbit, 1:500, Cell signalling, 3031S); GFP (Rabbit, 1:1000 Santa Cruz, sc-8433) UBF (Mouse, 1:500, Santa Cruz, sc-13125); UBF^S484^ (Mouse. Assaybiotech, A8444); p53 (Mouse, 1:2000; Oncogene OP43); IκB (Sheep, 1:5000, gift from RT Hay, University of Dundee). Cell cycle was analysed used FACs analysis of fixed, DAPI stained cells as previously described (28).

### Immunoprecipitation

Immunoprecipitation assays were performed using Magnetic beads (Dynabeads®, Novex) and 1mg whole cell lysate, prepared in NP40 lysis buffer. Mouse TIF-IA antibody (Santa Cruz Biotechnology) was used to immunoprecipitate the appropriate protein. Mouse IgG (pre-immune serum) acted as a control. Complexes were resolved by SDS polyacrylamide gel electrophoresis then analysed by western blot analysis.

### Phosphopeptide mapping

TIF-IA phosphorylation status was analysed by mass spectrometry as previously described (29). Briefly, SW480 cells were treated with aspirin (0 or 10 mM, 2 h) then pelleted cells disrupted in NP40 buffer and TIF-IA immunoprecipitated as above. Tryptic peptides were generated by on bead digestion and analysed on a Q-Exactive mass spectrometer connected to an Ultimate Ultra3000 chromatography system (both Thermo Scientific, Germany). Mass spectra were analysed using the MaxQuant Software package in biological triplicate and technical replicate. The abundance of phosphopeptides was determined as the ratio obtained by dividing the intensity of phosphopeptides by the intensity of the corresponding non-phosphorylated peptide.

### *Ex vivo* treatment of tumour biopsies and immunohistochemistry

Biopsies of colorectal tumours were provided by a pathologist at the time of resection. All patients were consented and full ethical approval was in place (Scottish Colorectal Cancer Genetic Susceptibility Study 3; Reference: 11/SS/0109). Biopsies were immediately transferred to the lab immersed in culturing media (MEM supplemented with glutamine, penicillin/streptomycin and anti-mycotic/antibiotic mix (1:100, Sigma). Tumours were washed, dissected into 1–2 mM fragments then plated. Treatment (0–100 μM aspirin, 1 h, 37°C) of tumour explants was performed in 96-well plates in duplicate in the presence of 10% foetal calf serum (30). Following treatment, tumours were either frozen for protein analysis (set 1) or formalin fixed for immunohistochemistry (set 2). Whole cell extracts were prepared using a TissueLyser (Qiagen) and standard whole cell lysis buffer.

Anti-^p536^RelA immunohistochemistry was carried out using a DAB protocol on formalin fixed sections, as previously described (31). A Leica scanner digitised images then Leica QWin plus image analysis software (Leica Microsystems Inc., Buffalo, IL, USA) used to analyse cells for nuclear RelA^p536^ staining. Three distinct areas of tissue and at least 1500 cells were analysed per section (average 4650). Scripts written in house determined the percentage of cells showing negative, weak, moderate and strong RelA^p536^ staining.

### Statistical analysis

*P* values throughout were calculated using a two-tailed Student's *t*-test unpaired with equal variance. For immunocytochemistry, the mean of at least five fields of view was determined for each condition then *P* values generated using the mean from at least three independent experiments (or as specified in the figure legend). *P* values in Figure 8D were derived using data from all cells analysed. For quantitative PCR, *P* values were generated by comparing the mean of three technical repeats, for three individual experiments, or as specified in the figure legend. *P* values for reporter assays represent the mean of at least 3 biological repeats. Pearsons correlation coefficient (r^2^) was used to examine the relationship between TIF-IA degradation and nuclear intensity of RelA in patient samples.

## RESULTS

### Silencing of PolI complex components activates the NF-κB pathway

**Figure 1.**
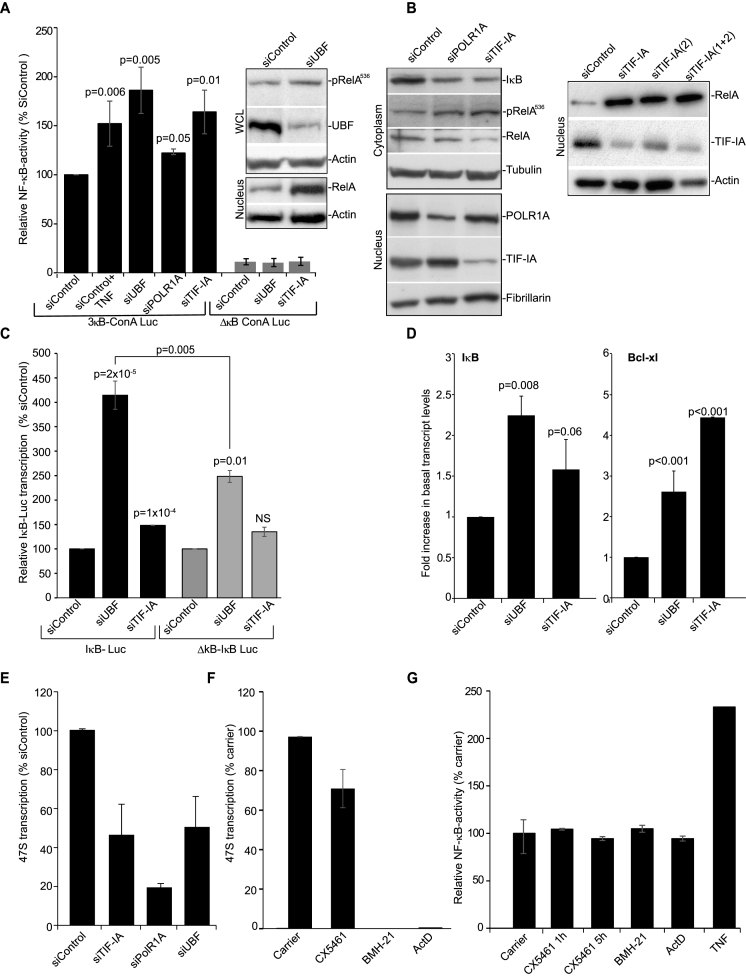
Silencing PolI complex components stimulates the NF-κB pathway. (A–C and E) SW480 cells were transfected with the indicated siRNA species. (**A**) Cells were co-transfected with pCMVβ and either a wild-type NF-κB-dependent luciferase reporter construct (3× κB ConA) or an equivalent plasmid with κB sites deleted (ConAΔκB). TNF (10 ng/ml, 4 h) acts as a control NF-κB stimulant. Luciferase activity was normalized using β-galactosidase activity. Results are presented as the percentage of relative NF-κB activity compared to cells transfected with 3× κB ConA-Luc/control siRNA (siControl). The mean of at least 3 repeats (±s.e.m.) is shown. Inset: Levels of pRelA^536^, nuclear RelA and UBF in whole cell (WCL) or nuclear lysates were determined upon UBF silencing using Western blot analysis. Actin acts as a control. (**B**) Immunoblots demonstrate- Left: reduced cytoplasmic IκBα, increased pRelA^S536^ and decreased cytoplasmic RelA upon silencing of TIF-IA and POLR1A. Nuclear extracts confirm efficient protein depletion. Right: increased nuclear RelA upon depletion of TIF-IA by two independent siRNAs. α-Tubulin, fibrillarin and actin act as loading controls. (**C**) Cells were co-transfected with IκB-luc (luciferase driven by full length IκB promoter) or ΔκB-IκB-luc (equivalent in which NF-κB sites are deleted) and pCMVβ. The percentage relative luciferase activity compared to siControl was calculated. Mean ± s.e.m. is shown (*N* = 3). (**D**) HCT116 cells were transfected as above. qRT-PCR was performed with primers for the NF-κB target genes IκBα and Bcl-xl. GAPDH was used to normalise. Results are presented as the fold increase in transcript compared to siControl. The mean (± s.e.m.) is shown. *N* = 3. (**E**) qRT-PCR with primers for the 47S pre-rRNA transcript measured levels of rRNA transcription. GAPDH was used to normalise. Results are presented as the percentage of relative 47S transcription compared to siControl. The mean (± s.e.m.) is shown. *N* > 3 (**F**) SW480 cells were treated with the PolI inhibitors CX5461 (500 nM), BMH-21 (4 uM), ActinomycinD (ActD, 1 ug/ml) or TNF (10 ng/ml), for 5 h. qRT-PCR measured levels of the 47S transcript as above. Mean ± s.e.m. is shown (*N* = 2). (**G**) SW480 cells were transfected with 3× κB ConA-Luc and pCMVβ. Twenty-fours hours later they were treated with inhibitors as in F. Graph shows the mean of at least two individual repeats ±s.e.m. *P* values throughout are compared to the respective control and were derived using a two tailed Student's *t* test. *N* values throughout are biological repeats. In (C), the *P* value for siUBF IκB-Luc versus siUBF ΔκB-IκB-Luc is also given. See Supplemental Figure S1 for additional cell lines and supporting data.

To investigate the link between nucleolar perturbation and NF-κB, we firstly inactivated an essential component of the PolI pre-initiation complex, upstream binding factor (UBF), then examined NF-κB pathway activity. This approach mimics that used by Rubbi and Milner to show nucleolar stress stabilizes p53 (5). We found siRNA silencing of UBF caused an increase in S536 phosphorylated RelA (a marker for cytoplasmic activation of the NF-κB pathway), a significant increase in nuclear RelA, and an increase in NF-κB-driven transcription comparable to that observed when cells were treated with the classic NF-κB stimulus, TNF (Figure 1A). Degradation of IκBα, pRelA^S536^ phophorylation and increased NF-κB-driven transcription were also observed upon silencing of the PolI components, POLR1A (RPA194) and TIF-IA (Rrn3p) (Figure 1A and B). Immunoblot analysis confirmed that depleting TIF-IA, using two independent siRNAs, induces nuclear accumulation of RelA (Figure 1B).

A link between PolI complex disruption and increased NF-κB activity was further confirmed using independent cell lines (Supplemental Figure S1A and C) and an independent reporter plasmid in which transcription of the luciferase gene is driven by the full-length promoter of the classic NF-κB target, IκBα (Figure 1*C)*. Indeed, depletion of UBF caused a >5-fold increase in transcription from the IκBα promotor. This effect was apparent, but significantly reduced when an equivalent reporter plasmid lacking κB sites was utilised (Figure 1C). These data suggest that the effect of PolI complex disruption on IκBα transcription is predominantly driven by NF-κB, although other factors may play a role. qRT-PCR confirmed that silencing UBF or TIF-IA induces transcription of IκBα and an independent NF-κB target, Bcl-xl (Figure 1D and Supplemental Figure S1B).

Given that disrupting nucleoli is linked with dramatic changes in the nucleoplasmic proteome (3), we did consider that silencing PolI complex components may stimulate NF-κB-driven transcription in the absence of IκB degradation/cytoplasmic release of NF-κB. However, the significant increase in NF-κB-driven transcription observed in control cells upon depletion of UBF and TIF-IA was blocked in cells we generated to constitutively express super repressor (non-degradable) IκBα (Supplemental Figure S1C). Hence, we conclude that IκBα degradation is an essential step for nucleolar stress to enhance NF-κB transcriptional activity.

qRT-PCR for the 47S pre rRNA transcript confirmed that siRNA to UBF, POLRIA and TIF-IA inhibited rDNA transcription (Figure 1E and Supplemental Figure S1D). Stabilization of p53 following nucleolar stress is strongly linked to inhibition of rDNA transcription (6). Therefore, we considered this may also be the case for activation of NF-κB. However, actinomycinD and two highly specific small molecule inhibitors of PolI (CX5461 and BHM-21 (32,33) had no effect on NF-κB transcriptional activity, while causing a significant reduction in levels of the 47S transcript (Figure 1F and G).

Taken together, these data suggest that it is not inhibition of rRNA transcription *per se*, but a specific type of perturbation of the PolI complex that activates the cytoplasmic NF-κB pathway. To test this suggestion, we next examined the effects of NF-κB stress stimuli on this complex.

### Degradation of TIF-IA precedes NF-κB pathway activation

TIF-IA is essential for rDNA transcription as it tethers Pol I to the rDNA promoter (34,35). It is also *the* component of the complex that is targeted by stress/environmental signals to alter PolI activity (36,37). Therefore, we firstly investigated this protein. Aspirin was initially chosen as a model stimulus as we are interested in the pro-apoptotic activity of this agent and in the absence of additional cytokines, it stimulates NF-κB in a manner characteristic of multiple stress inducers (14,26).

**Figure 2.**
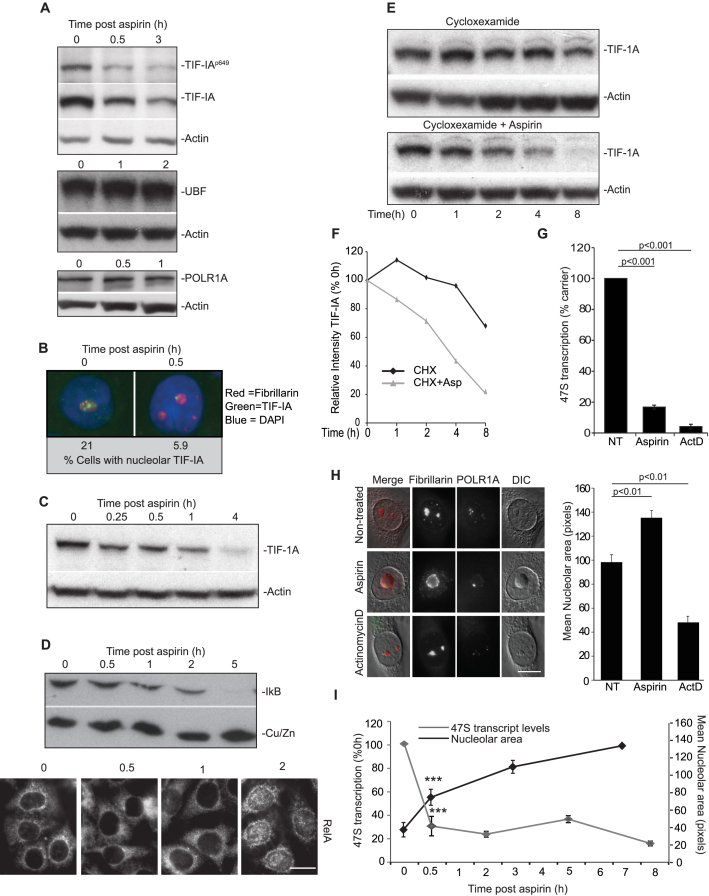
Degradation of TIF-IA and a distinct nucleolar phenotype precede aspirin effects on the NF-κB pathway. (**A**–**D**) Aspirin (used as a model stress stimuli) induces a decrease in total cellular levels of TIF-IA, which precedes degradation of IκB and nuclear accumulation of RelA. SW480 cells were treated with aspirin (10mM) for the indicated times (A, C and D) Immunoblot analysis was performed on WCL with the indicated antibodies. (B) Immunomicrographs (63X) show levels and localisation of native TIF-IA. Below: The percentage of nuclei (as depicted by DAPI stain) with bright puncti of TIF-IA were quantified using ImageJ software. A minimum of 200 nuclei were analysed per experiment from at least 10 fields of view. *N* = 3. (D) Bottom: Immunomicrographs (×63) demonstrate accumulation of RelA in nuclei 2 h after aspirin exposure. (**E** and **F**) SW480 cells were treated with cyclohexamide (10 uM) alone or with aspirin (10 mM) for the times specified. (E) Immunoblot shows cellular levels of TIF-IA. (F) TIF-IA band intensities relative to actin were quantified using ImageJ. Results are presented as the percentage compared to the 0 h control. One representative experiment is shown. *N* = 2. (**G**–**I**) Perturbation of nucleolar structure and function in response to aspirin. (G) rDNA transcription was quantified after aspirin (3 mM, 16 h) or actinocycinD (50 ng/ml, 2 h) treatment using qRT-PCR for the 47S transcript as above. Results are presented as the percentage of relative transcription compared to non-treated (NT) control. The mean (±s.e.m.) is shown. *N* = 4. (H) Representative DIC immunomicrographs (x63) showing the cellular localisation of components of the tripartite nucleolar structure in response to aspirin (10 mM, 8 h) and actinomycinD (50 ng/ml). Fibrillarin marks the dense fibrillar component and POLR1A the PolI complex in the fibrillar centre. Nucleolar area was quantified using ImageJ and fibrillarin staining (to define nucleoli). At least 250 cells were analysed per experiment. Graph depicts the mean (±s.e.m.) of three experiments. (I) rDNA transcription and nucleolar size were monitored over time in SW480 cells using qRT-PCR for the 47S transcript (as above) and ImageJ analysis of area devoid of DAPI staining (as a marker for nucleoli). At least 200 cells from 10 fields of view were analysed for nucleolar area. Graph depicts the mean of three experiments (±s.e.m). ***P < 0.001. Actin and Cu/ZnSOD act as loading controls throughout. Scale bars = 10 μm. *P* values throughout are compared to the respective control and were derived using a two tailed Student's *t* test. N values are biological repeats. See Supplemental Figure S2 for additional cell lines and supporting data.

Figure 2A demonstrates that aspirin not only induces a decrease in Ser649 phosphorylated TIF-IA, which is a known response to environmental stress, but also a significant decrease in native TIF-IA, which is *not* a reported stress response (Figure 2A). This decrease in native TIF-IA was observed by Western blot analysis (Figure 2A) and by immunocytochemistry (Figure 2B). It was evident in multiple cell types, was independent of p53 status and most importantly, was an early response to the agent, preceding degradation of IκB and nuclear translocation of RelA (Figure 2C and D and Supplementary Figure S2A to C) (22,26).

qRT-PCR and cyclohexamide run on assays indicated that the aspirin-induced reduction in TIF-IA was not a consequence of reduced gene transcription, but caused by increased protein turnover (Figure 2E and F and Supplemental Figure S2D). Neither proteasome nor lysosome inhibitors alone could block this increased turnover, which was only abrogated when inhibitors of both pathways were combined (Supplemental Figure S2E–H). Nutlin-3, the MDM2 inhibitor known to block basal TIF-IA turnover, actually enhanced aspirin-mediated TIF-IA degradation, suggesting it takes place by an unreported mechanism (Supplemental Figure S2E) (15). Exogenously expressed TIF-IA was also rapidly degraded in response to aspirin (Supplemental Figure S2I). In contrast, the agent had no effect on levels of UBF or POLR1A (Figure 2A).

Classic hallmarks of nucleolar stress are inhibition of rDNA transcription, segregation of nucleolar marker proteins and reduced nucleolar area (2). Given the role of TIF-IA in maintaining nucleolar structure (38), we investigated these hallmarks. We found aspirin-mediated TIF-IA degradation was associated with a significant decrease in rDNA transcription, as indicated by 47S qRT-PCR and 5-fluorouridine (FUrd) run on assays (Figure 2G and Supplemental Figure S3A). It was also associated with nucleolar segregation, as evidenced by relocation of all three components of the tri-partite nucleolar sub-structure to the periphery of the organelle (Figure 2H and Supplemental Figure S3B). However, in contrast to actinomycinD which caused a significant *reduction* in nucleolar area, aspirin induced a significant *increase* in nucleolar area (Figure 2H). This increase was an early response to the agent, paralleling degradation of TIF-IA and inhibition of rDNA transcription (Figure 2C and I and Supplemental Figure S2C).

### Generality of TIF-IA degradation in response to stress stimuli of the NF-κB pathway

**Figure 3.**
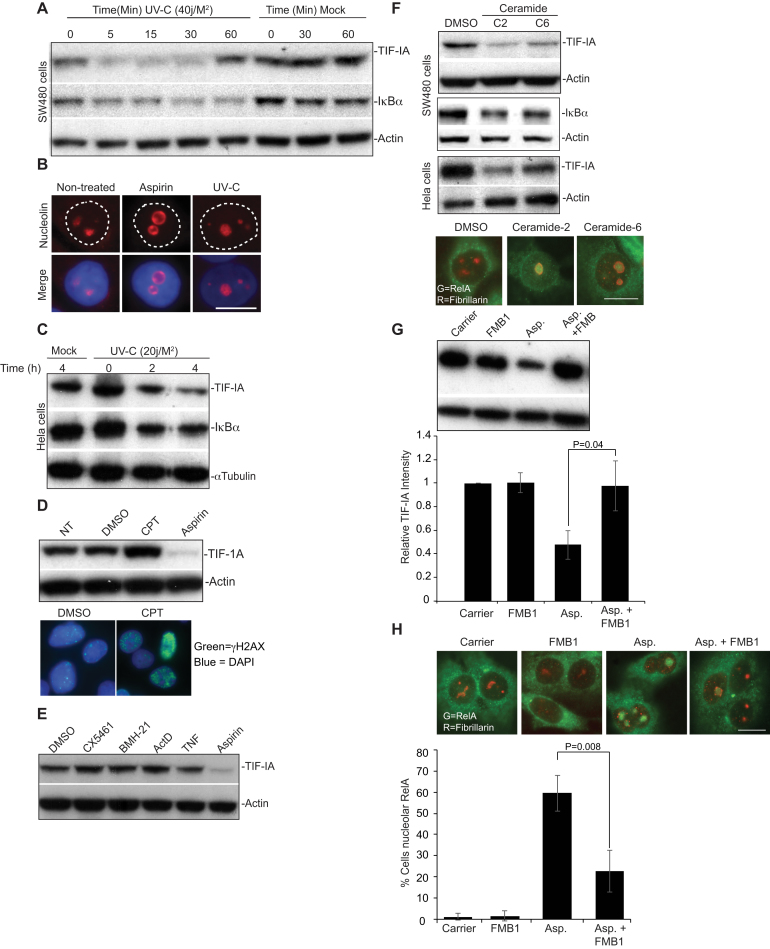
TIF-IA degradation in response to multiple stress stimuli of NF-κB (**A** and **C**) SW480 or Hela cells were mock or UV-C (20 or 40 J/m^2^) irradiated. Following the times specified, immunoblots was performed on WCL with the indicated antibodies. (**B**) Immunomicrograph (×63) showing increased nucleolar area (as depicted by nucleolin staining) in SW480 cells in response to aspirin (3 mM, 16 h) or UV-C (40 J/m^2^, 2 h). (**D**) SW480 cells were treated with carrier, 10 μM Camptothecin (CPT) or aspirin (3 mM) for 16 h. *Top:* Western blot was performed with the indicated antibodies on WCL. *Bottom*: γH2AX immunocytochemistry confirmed DNA damage in response to CPT. (**E**) SW480 cells were treated with DMSO (carrier), CX5461 (500 nM), BMH-21 (4 μM), ActinomycinD (ActD, 50 ng/ml) or aspirin (10 mM) for 4 h, or TNF (10 ng/ml) for 30 min, WCL were examined by western blot using the antibodies indicated. (**F**) SW480 or Hela cells were treated with carrier (DMSO), ceramide-2 (C2, 10 uM)) or ceramide-6 (C6, 10 uM) for 16 h. *Top:* WCL were analysed by western blot with the indicated antibodies. *Bottom:* Representative immunomicrographs (×63) show the localisation of fibrillarin and RelA in SW480 cells. (**G**) SW480 cells were pre-treated with 100 μM FumonisinB1 (FMB1), prior to aspirin (3 mM,16 h) exposure. *Top:* Immunoblots indicate cellular levels of TIF-IA. TIF-IA intensity (relative to actin) was determined for each condition using ImageJ analysis. Results are presented as the percentage relative TIF-IA compared to control. Mean (±s.e.m.) is shown for six experiments. Bottom: Representative immunomicrograph (×63) demonstrating the cellular localisation of RelA. Fibrillarin acts as a nucleolar marker. The percentage of cells showing nucleolar RelA was determined manually. At least 200 cells from at least 5 fields of view per were counted per experiment. The results are the mean (±s.e.m.). *N* = 3. P values were derived using a two tailed Student's t test. Scale bars = 10 μm. Actin or α tubulin act as loading controls.

Having established aspirin has a unique effect on TIF-IA and nucleolar structure, we next examined the generality of this response with regards to stress stimuli of NF-κB. We found that, like aspirin, UV-C induced a rapid depletion of TIF-IA (Figure 3A–C). Although this depletion was transient in SW480 cells, it still preceded degradation of IκB and was paralleled by enlargement of nucleoli (Figure 3A–C). In contrast to UV-C, the DNA damaging agent camptothecin, which has previously been shown to cause nucleolar stress (Supplemental Table S1), actually increased cellular levels of TIF-IA (Figure 3D). It also had a minimal effect on nucleolar structure (Figure 3D). Similarly, actinomycinD, BMH-21, CX5461 and TNF did not significantly alter TIF-IA levels (Figure 3E).

Aspirin and UV-C generate ceramide, a crucial lipid second messenger that is a potent stimulus of the NF-κB pathway (39–41). When we tested the C2 and C6 soluble forms of ceramide, which mimic the lipid increase observed in response to stress, we found that they also induced degradation of TIF-IA (Figure 3*F*). Furthermore, this occurred in association with increased nucleolar area, degradation of IĸB and nucleolar translocation of RelA (Figure 3*F*). To further explore the role of ceramide we utilised the ceramide synthase inhibitor, FumonisinB1. Figure 3G and H demonstrate that exposure to this inhibitor not only abrogates aspirin-mediated degradation of TIF-IA, but also significantly reduces nucleolar translocation of RelA.

Together, these data suggest that specific stresses target TIF-IA for degradation through a novel, ceramide dependent pathway, and that this degradation causes distinctive changes in nucleolar structure and activation of NF-κB. To explore this possibility, we set out to further elucidate the mechanism underlying stress-mediated TIF-IA degradation.

### CDK4 inhibition mimics stress effects on TIF-IA and nucleoli

A common early response to aspirin, UV-C and ceramide is reduced activity of the cyclin dependent kinase, CDK4 (42). Since CDK4 targets components of the PolI complex, we considered that reduced activity of this kinase may play an important upstream role. To test this possibility, we mimicked stress effects on CDK4 using a highly specific, small molecule inhibitor (CDK4i), then monitored downstream consequences on TIF-IA and nucleoli.

**Figure 4.**
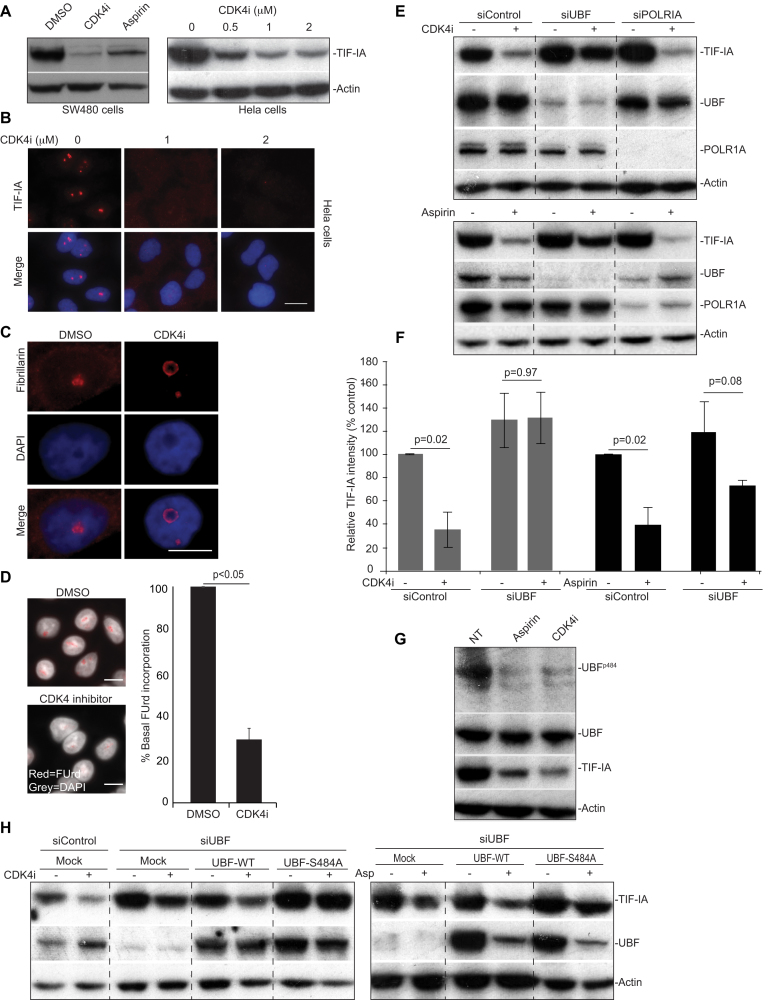
A role for CDK4 and UBF S484 in TIF-IA degradation. (**A**–**D**) CDK4 inhibition induces degradation of TIF-IA and atypical changes to nucleolar structure. SW480 or Hela cells were treated with DMSO (carrier), aspirin (3 mM, 16 h) or the small molecule CDK4 inhibitor, 2-bromo-12,13-dihy-dro-indolo[2,3-*a*]pyrrolo[3,4-*c*] carbazole-5,7(6*H*)-dione (CDK4i, 2 uM or as indicated). (A) Anti-TIF-IA immunoblot performed on WCL. (B) Immunomicrographs (63×) demonstrating the levels and localisation of TIF-IA in Hela cells. (C) Immunomicrograph (63X) demonstrating re-localisation of fibrillarin in response to CDK4i in SW480 cells. (D) *Left:* Immunomicrographs (40×) depicting cells subjected to fluouridine (FUrd) run on assays. *Right:* Images were captured and analysed for FUrd incorporation using ScanR image analysis software. The results are presented as the percentage incorporation compared to control. The mean (±s.e.m.) of at least 1000 cells per experiment is shown. N=3 (**E** and **F**) SW480 cells were transfected with control, UBF or POLRIA siRNA. Forty-eight hours later cells were treated (+) with CDK4i (2 uM, 16 h), aspirin (3 mM 16 h) or the equivalent carriers (–). (E) Western blot analysis was performed with the indicated antibodies. (F) TIF-IA intensity (relative to actin) was determined for each condition using ImageJ analysis. Results are presented as the percentage relative TIF-IA compared to carrier treated, siControl. Mean (± s.e.m.) is shown for 2 (CDK4i) and 3 (aspirin) experiments. (**G** and **H**) Identification of a role for residue 484 of UBF. (G) SW480 cells were treated with aspirin and CDK4i as above. Western blot analysis was performed with antibodies to phosphorylated (UBF S484) and native UBF. (H) SW480 cells were transfected with control or UBF siRNA then either mock transfected or transfected with Flag-UBF-wild type (WT) or a phospho-mutant-flag-UBFS484A. Eight hours later, transfected cells were treated with CDK4i and aspirin (asp.) as above. Immunoblot was performed with the indicated antibodies. Scale bar = 10 μm. Actin acts as a loading control throughout. *P* values were derived using a two tailed Student's *t* test. See also Supporting Supplemental Figure S3.

Immunoblot and immunocytochemical analysis revealed that exposure of cells to CDK4i induced a substantial, dose dependent reduction in TIF-IA protein levels (Figure 4A and B). This reduction was evident in multiple cell types (Figure 4A). It was also observed in response to an independent CDK4 inhibitor, Palbociclib (PD0332991) (Supplementary Figure S4A) and to siRNA depletion of the protein (Supplementary Figure S4B). Furthermore, it was paralleled by the same distinct nucleolar phenotype as stress stimuli of the NF-κB pathway i.e increased nucleolar size, segregation of nucleolar marker proteins and inhibition of rDNA transcription (Figure 4C and D). Like aspirin, CDK4i-mediated TIF-IA degradation was blocked by proteasome and lysosomal inhibitors and enhanced in the presence of nutlin-3, suggesting it is mediated through the same distinctive pathway (Supplementary Figure S4C and D). In contrast to CDK4 inhibition, Roscovitine, which inhibits CDK1, CDK2 and CDK5 (but not CDK4/6), had a minimal effect on TIF-IA levels (Supplementary Figure S4E).

We did consider that stress effects on TIF-IA may be linked to cell cycle, although this did seem unlikely given that TIF-IA degradation was evident minutes after aspirin/UV-C exposure (Figures 2A and 3A). Furthermore, aspirin and Roscovitine arrest cells in the same phase of the cell cycle (G2/M), but have opposing effects on TIF-IA, while CDK4i inhibits cells in G1, but has the same effect as aspirin (Supplemental Figure S4E and F). Therefore, we concluded that TIF-IA degradation is not a consequence of stress-mediated cell cycle arrest, but a specific response to CDK4 inhibition.

### Identification of a role for UBF S484 and p14ARF in TIF-IA degradation

CDK4 regulates rDNA transcription by targeting UBF (43). Therefore, we considered UBF may be required for TIF-IA degradation downstream of CDK4 inhibition. Indeed, siRNA silencing of UBF significantly abrogated CDK4i-mediated TIF-IA degradation (Figure 4E and F). Silencing of UBF also abrogated TIF-IA degradation in response to the model stress inducer, aspirin (Figure 4E and F). In contrast, silencing POLR1A did not perturb TIF-IA degradation in response to either agent, suggesting specificity (Figure 4E).

CDK4 phosphorylates UBF on Serine 484 and so, we next explored the role of this residue (43). Figure 4G demonstrates that both aspirin and CDK4i cause a decrease in UBF S484 phosphorylation, in keeping with inhibition of the CDK4 kinase. To determine whether this decrease is essential for TIF-IA degradation we utilised a mutant that cannot be phosphorylated at this site (Flag-UBF S484A). SW480 cells were depleted for UBF then transfected with plasmids expressing either wild type protein (Flag-UBF-WT) or the Flag-UBF-S484A mutant. If de-phosphorylation of UBF at S484 was important for TIF-IA degradation, we would expect that the phospho-mutant would mimic the effects of CDK4i on TIF-IA, or at least enhance CDK4i-mediated degradation of the protein. However, contrary to this expectation, expression of flag-UBF-S484A actually blocked TIF-IA degradation in response to CDK4i and aspirin (Figure 4H). Again, we found that silencing of UBF abrogated CDK4i-mediated degradation of TIF-IA, while expression of wild type UBF rescued this effect (Figure 4H). Together, these data indicate a crucial role for UBF, and in particular residue 484, in stress-mediated degradation of TIF-IA. However, the fact that the phospho-mutant blocked TIF-IA degradation would suggest it is not dephosphorylation at this site that is important.

**Figure 5.**
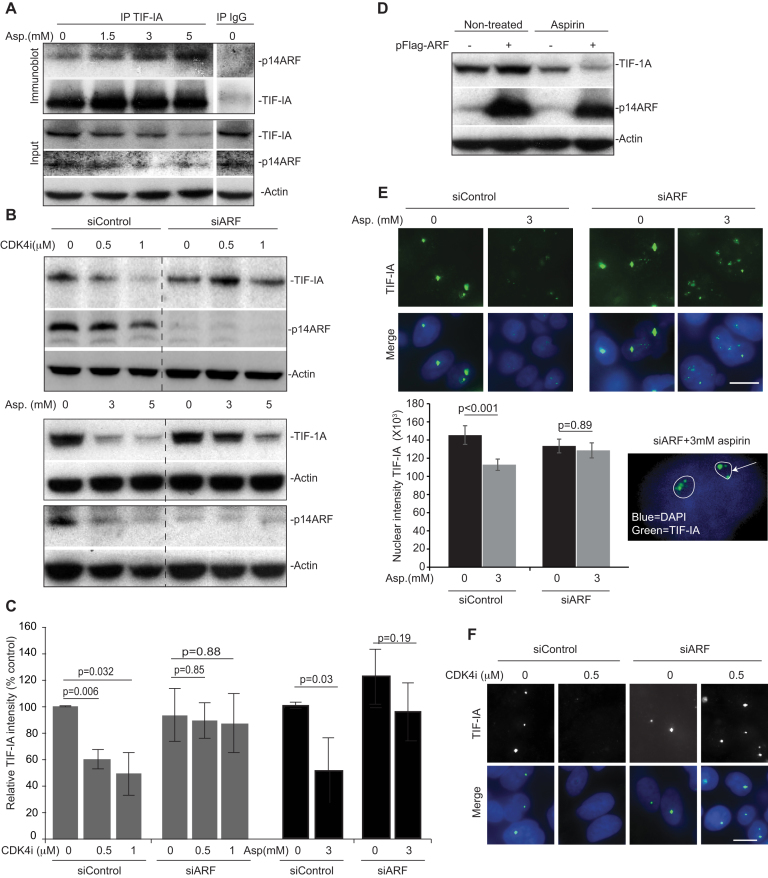
Identification of a role for p14ARF in TIF-IA degradation. (**A**) TIF-IA interacts with p14ARF in response to aspirin. SW480 cells were treated with 0–5 mM aspirin for 16 h. Immunoprecipitation was carried out on WCL using antibodies to TIF-IA and IgG control. Precipitated proteins were subjected to western blot analysis with the indicated antibodies. Input levels are shown. (**B**–**F**) Silencing of P14ARF is required for stress-mediated degradation of TIF-IA. (B, C, E and F) SW480 cells were transfected with control or p14ARF siRNA (siARF) then treated for 16 h with CDK4i (0–1 μM) or aspirin (0–3 mM). (B) Immunoblot analysis was performed on WCL with the indicated antibodies. (C) TIF-IA levels relative to actin were quantified using ImageJ analysis. Graph shows the mean (±s.e.m.) compared to non-treated, siControl. *N* = 3. (E and F) Immunomicrographs (×63) show the levels and localisation of TIF-IA in fixed cells. (E) IPlab software quantified nuclear (as depicted by DAPI staining) intensity of TIF-IA. Data are the mean (±s.e.m.) of >150 nuclei. *N* = 3. Inset shows nucleolar (outlined) TIF-A in p14ARF transfected cells treated with aspirin. (D) SW480 cells were transfected with pcDNA3 control plasmid (–) or pcDNA3-p14ARF (+) then either non-treated or treated with aspirin (3 mM, 16 h). Immmunoblot was performed on WCL with the indicated antibodies. Actin acts as a loading control throughout. Scale bar = 10 μm. N values are biological repeats. *P* values were derived using a two-tailed Student's *t* test. See also supporting Supplemental Figure S4.

p14ARF is a nucleolar tumour suppressor that regulates rRNA synthesis in a manner dependent upon S484 of UBF (44,45). It is also known to influence NF-κB signalling (46). Therefore, we considered it may play a role. Indeed, immunoprecipitation assays revealed that TIF-IA complexes with P14ARF in response to aspirin in a time and dose dependent manner that parallels degradation of the protein (Figure 5A and Supplementary Figure S5A). Furthermore, immunoblot analysis indicated that siRNA silencing of p14ARF blocks CDK4i and aspirin-mediated degradation of TIF-IA, while over-expression enhances this effect (Figure 5B–D). Aspirin alone did appear to cause a dose dependent reduction in p14ARF levels (Figure 5B). However, detailed time course studies revealed that TIF-IA degradation precedes loss of p14ARF (Supplementary Figure S5B). Immunocytochemical analysis confirmed that aspirin and CDK4i cause a significant reduction in nuclear TIF-IA in cells transfected with control siRNA, but not in cells transfected with p14ARF siRNA (Figure 5E and F). Interestingly, in the absence of p14ARF, TIF-IA remained within nucleoli following aspirin and CDKi exposure suggesting this protein may play a role in nucleolar export (Figure 5E and F).

Together, these data provide very strong evidence for a ceramide-CDK4-UBF/p14ARF stress response pathway that lies upstream of TIF-IA degradation in response to stress stimuli.

### Identification of a link between TIF-IA degradation and activation of the NF-κB pathway

**Figure 6.**
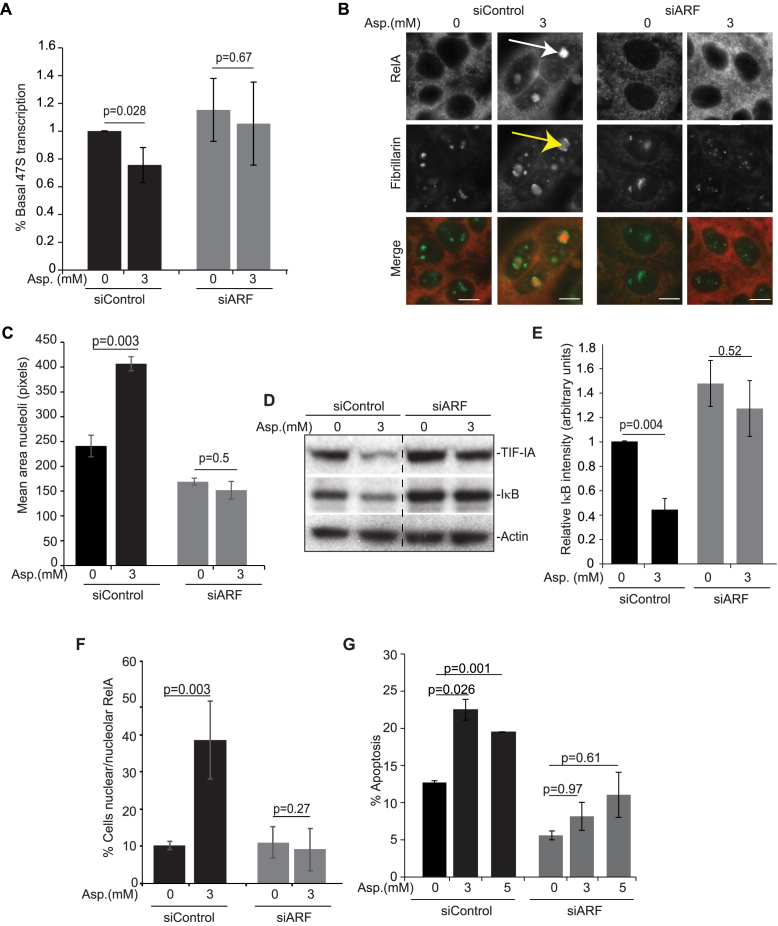
Blocking TIF-IA degradation inhibits aspirin effects on nucleoli and the NF-κB pathway. (**A**–**G**) Blocking TIF-IA degradation, using siRNA to p14ARF, abrogates aspirin-mediated inhibition of rDNA transcription, nucleolar enlargement, degradation of IκB, nuclear/nucleolar translocation of RelA and apoptosis. SW480 cells were transfected with control or p14ARF siRNA as in Figure 6 then treated with aspirin (Asp.) at the concentrations specified. (A) qRT-PCR with primers for the 47S pre-rRNA transcript measured levels of rRNA transcription. GAPDH was used to normalise. Results are presented as the percentage of relative 47S transcription compared to the equivalent 0mM control for each siRNA. Mean (±s.e.m.) of 3 is shown. (B) Immunocytochemistry was performed on fixed cells with the indicated antibodies. Arrows indicate nucleolar RelA (white) and enlarged, segregated nucleoli (yellow). (C) Nucleolar area was quantified in at least 150 cells using IPlab software with fibrillarin as a nucleolar marker. Mean (± s.e.m.) is shown. *N* = 3. (D) Immunoblots demonstrating cytoplasmic levels of IκBα. (E) ImageJ software measured IκBα intensity relative to actin. The results are the mean of 3 experiments ±s.e.m. (F) Immunocytochemistry was performed as in B. The percentage of cells in the population showing nucleolar RelA was quantified manually. At least 6 fields of view and >100 cells were analysed per condition. The mean ± s.e.m is shown. *N* = 3. (G) Annexin V apoptosis assays were performed. The percentage of cells undergoing apoptosis was determined by fluorescent microscopy. At least 200 cells were analysed for each sample. The results are the means of two independent experiments ± s.e.m. Actin acts as a loading control. Scale bar = 10 μm. *P* values were derived using a two-tailed Student's *t* test. N values are biological repeats.

**Figure 7.**
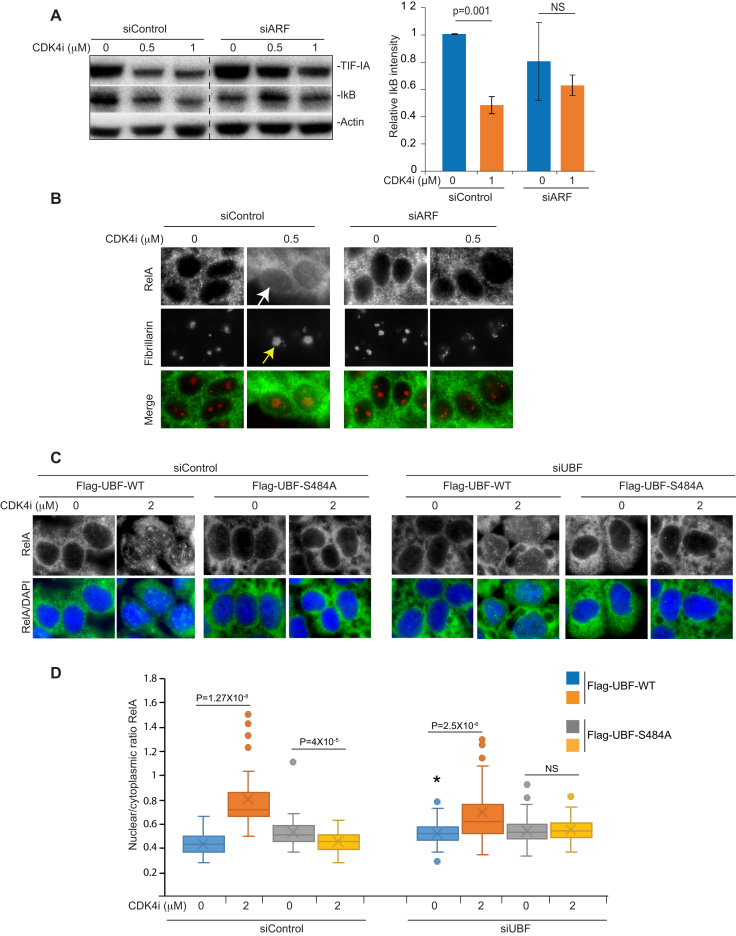
Blocking TIF-IA degradation inhibits CDK4i effects on nucleoli and the NF-κB pathway. (A and B) SW480 cells were transfected with control or p14ARF siRNA as above then treated with CDK4i at the concentrations specified. (**A**) Immunoblot demonstrating cytoplasmic levels of IκBα. Right: ImageJ software measured IκBα intensity relative to actin. The results are the mean of two experiments ± s.e.m. (**B**) Immunocytochemistry was performed on fixed cells with the indicated antibodies. Arrows indicate increased nuclear RelA (white) and nucleolar area (yellow) in response to CDK4i in siControl transfected cells. (**C**) SW480 cells were transfected with the indicated siRNA species alongside Flag-UBF WT or S484A (as in Figure 4H). Following CDK4i treatment (0–2 μM), immunocytochemistry was performed on fixed cells with antibodies to RelA. ImageJ was used to quantify the nuclear to cytoplasmic intensity of RelA. A Whisker plot shows nuclear/cytoplasmic ratios for at least 100 cells per condition per experiment (*N* = 2). Actin acts as a loading control throughout. Scale bar = 10μm. *P* values were derived using a two-tailed Student's *t* test. *P = 0.05 when compared to siControl.

Using p14ARF siRNA, we next questioned the role of TIF-IA degradation in stress effects on the NF-κB pathway and nucleolar structure. Figure 6A–C demonstrate that blocking TIF-IA degradation in this manner significantly abrogates aspirin-mediated inhibition of rDNA transcription and enlargement of nucleoli. Furthermore, the significant degradation of IκB and nuclear/nucleolar accumulation of RelA observed in response to aspirin in control siRNA transfected cells was blocked in cells transfected with siRNA to p14ARF (Figure 6B and D–F). Aspirin-mediated apoptosis was also abolished by silencing of p14ARF, which would be expected given our data showing NF-κB pathway activation is required for the apoptotic effects of the agent (26) (Figure 6G). Similar results were obtained with CDK4i in that p14ARF silencing abrogated CDK4i-mediated nucleolar enlargement, degradation of IκB and nuclear accumulation of RelA (Figure 7A and B).

Next, we utilised the UBF-S484A mutant to investigate the link between TIF-IA degradation and NF-κB signalling. SW480 cells were transfected with control or UBF siRNA prior to overexpression of Flag-UBF-WT or -S484A. Quantitative immunocytochemistry was then used to determine nuclear to cytoplasmic ratios of RelA. (Figures 7C and D). Figure 7D clearly demonstrates that the significant increase in nuclear RelA observed in cells expressing Flag-UBF-WT is blocked in cells expressing Flag-UBF-S484A (Figure 7C and D). Furthermore, it demonstrates that CDK4i has a greater effect on the NF-κB pathway in cells expressing the most UBF (control siRNA, Flag-UBF-WT), which would be expected if UBF is essential. Together, these data reveal a new pathway by which stresses act on the PolI complex that involves CDK4-UBF(S484)/p14ARF facilitated degradation of TIF-IA. They also suggest atypical changes in nucleolar architecture and activation of the NF-κB pathway are downstream consequences of this specific PolI complex disruption.

### Dephosphorylation of S44 is critical for stress-mediated degradation of TIF-IA and nuclear translocation of RelA

**Figure 8.**
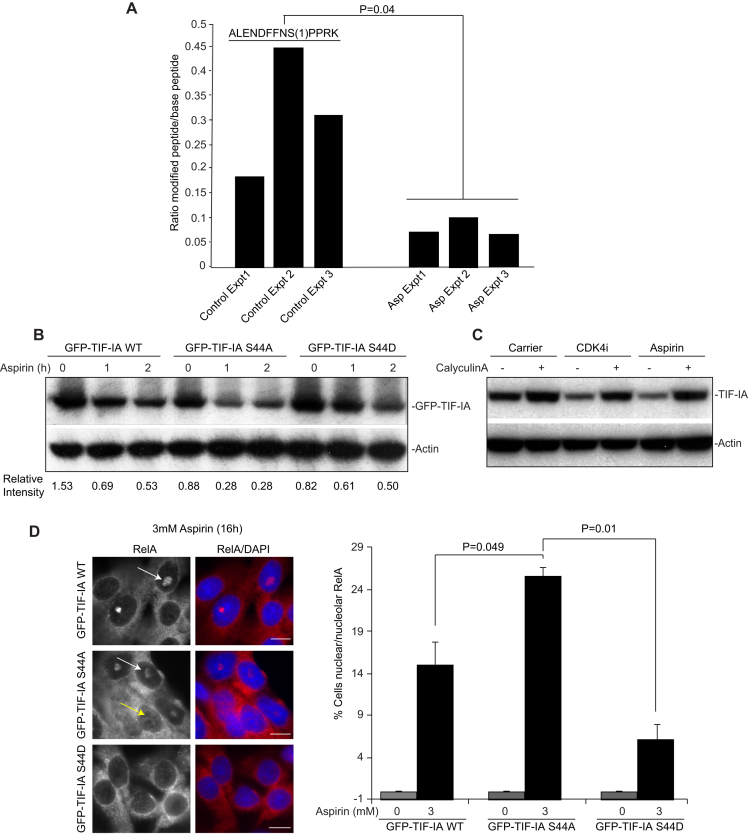
Dephosphorylation at S44 is critical for stress-mediated TIF-IA degradation and activation of the NF-κB pathway. (**A**) Label-free quantitative mass spectrometry (MaxLFQ), performed on immunoprecipitated endogenous protein, was used to screen for changes in TIF-IA phosphorylation in response to aspirin (0 or 10 mM, 2 h). Bar graph represents the ratio of phosphorylated to de-phosphorylated peptide in the presence and absence of aspirin, for three independent experiments. The peptide is shown, with the localisation of the phosphorylation site in brackets. (**B**) SW480 cells were transfected with the indicated GFP-TIF-IA mutants then treated with aspirin (0 or 10 mM) for the times specified. Immunoblots were performed on WCL with the indicated antibodies. The intensity of TIF-IA relative to actin is shown for one representative experiment (*N* = 3). (**C**) SW480 cells were pre-treated with CalyculinA (5 nM) for 4 h prior to aspirin (10 mM) or CDK4i (4 μM) exposure (4 h). Immunoblots were performed on WCL with the indicated antibodies. (**D**) SW480 cells were transfected with the indicated GFP-tagged plasmids then treated with aspirin (0 or 3 mM, 16 h). (Left) Immunomicrographs (×63) demonstrate the localisation of RelA in aspirin treated cell populations. DAPI staining depicts nuclei. (Right) The percentage of cells in the population showing nuclear (yellow arrow) or nucleolar (white arrow) RelA was quantified manually. Data are the mean of at least five fields of view (>150 cells), for two independent experiments (±s.e.m.). Bars, 10 μm. *P* values were derived as above.

**Figure 9.**
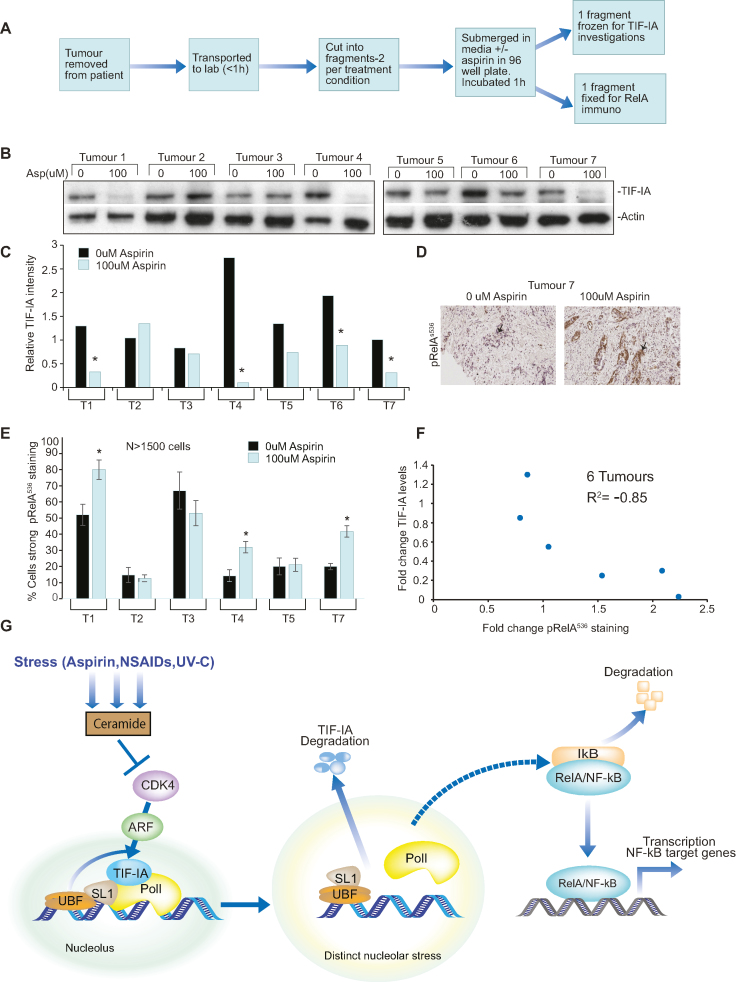
TIF-IA degradation correlates with NF-κB pathway activation in human clinical samples. (**A**) Diagram depicting workflow of *ex vivo* culture. Resected colorectal tumour biopsies were immediately transferred to the lab, washed, immersed in culturing media in 96-well plates then exposed to 0 or 100 uM aspirin for 1 h. One piece of tissue was fixed for immunohistochemistry while another was frozen for protein analysis. This was carried out for seven patients. (**B**) Immunoblot was performed on WCL with the indicated antibodies. (**C**) ImageJ quantified TIF-IA intensity relative to actin. * Tumours showing a >2-fold decrease in relative levels of TIF-IA in response to aspirin were deemed to respond. (D and E) Immunohistochemistry was performed on sections from paraffin embedded tissue with antibodies to RelA^p536^. (**D**) An example immunomicrograph. Arrows indicate epithelial cells. (**E**) Leica QWin plus image analysis software, with scripts written in house, was used to quantify the nuclear RelA^p536^ intensity in digitized images. Three distinct areas of tissue and at least 1500 cells were analysed per section. Data presented are the % cells showing moderate+strong RelA^p536^ staining, as indicated by image analysis software. * Significant (*P* < 0.05) difference between the % stained cells in treated and non-treated sections. (**F**) Graph showing the relationship between aspirin-induced changes in TIF-IA and RelA^p536^ staining for six individual tumours. Pearsons correlation coefficient (*r*^2^) was used to examine the relationship. (**G**) Proposed model. Generation of ceramide by specific stresses inhibits CDK4 kinase activity, which induces degradation of TIF-IA in a manner dependent on UBF/p14ARF. The consequent disruption of the PolI pre-initiation complex causes distinct changes in nucleolar architecture and triggers a protein or pathway (as yet unknown) which causes phosphorylation and degradation of IκBα, nuclear translocation of RelA and ultimately, transcription of a gene programme that alters cell phenotype. We propose the nature of this transcriptional program is cell type and stimulus dependent.

To provide further evidence for a direct relationship between TIF-IA degradation and activation of NF-κB, we next used label-free quantitative mass spectrometry (MaxLFQ), performed on immunoprecipitated endogenous protein, to identify post-translational modifications specifically altered by aspirin. Quantification of phosphorylation sites by LFQ revealed that serine 44 of TIF-IA is consistently phosphorylated in control samples, and that this phosphorylation is significantly reduced in response to the agent (Figure 8A). To understand the significance of this de-phosphorylation with respect to TIF-IA degradation, GFP-TIF-IA mutants were generated in which S44 is mutated to alanine (A) (which mimics de-phosphorylation) or aspartic acid (D) (which mimics phosphorylation). Figure 8B demonstrates that, compared to wild type protein, GFP-TIF-IA S44A shows enhanced degradation in response to aspirin while degradation of GFP-TIF-IA S44D is abrogated. Exposure to the phosphatase inhibitor, calyculinA, also blocked aspirin and CDK4i-mediated TIF-IA degradation (Figure 9C). These data confirm a critical role for S44 dephosphorylation in TIF-IA degradation, and suggest that protein phosphatases are critical in this process.

Next we used the TIF-IA S44 mutants to determine whether modulating TIF-IA degradation alters stress effects on the NF-κB pathway. SW480 cells were transfected with GFP-TIF-IA-WT, -S44A or -S44D, then quantitative immunocytochemistry used to analyse aspirin-mediated nuclear/nucleolar translocation of RelA. As aspirin induces degradation of GFP-TIF-IA prior to effects on NF-κB signalling, we could not do this analysis on transfected cells only. Therefore, we quantified the whole cell population. These data revealed that nuclear/nucleolar translocation of RelA was significantly enhanced in populations transfected with GFP-TIF-IA S44A, and significantly abrogated in those transfected with TIF-IA S44D (Figure 8D). The number of transfected cells prior to treatment, and the levels of expressed protein, were similar for all constructs, suggesting this difference was not a consequence of differential transfection efficiency (Figure 8B).

At this time, we cannot establish a link between UBF/p14ARF and dephosphorylation of TIF-IA at S44. Nonetheless, these studies reveal a critical role for TIF-IA S44 in the degradative response to stress and importantly, provide direct evidence for a link between TIF-IA degradation and activation of the NF-κB pathway.

### Relationship between TIF-IA degradation and stimulation of NF-κB signalling in human clinical samples

Overwhelming evidence indicates that aspirin has anti-tumour activity and the potential to prevent colorectal and other cancers (47,48). To investigate the clinical significance of our results with regards to this activity, and to determine whether there is a link between PolI complex disruption and NF-κB pathway activation in a whole tissue setting, we treated biopsies of fresh, surgically resected human colorectal tumours with pharmacological doses (0–100 μM, 1 h) of aspirin *ex vivo* (Figure 9A). This aspirin concentration is comparable to salicylate levels we measured in plasma from patients given a short course of analgesic doses of aspirin (26). It is also well within the reported therapeutic range (0.1–3 mM).

Western blot analysis revealed low dose aspirin induces TIF-IA degradation (as defined by a >2-fold reduction in protein levels) in 4/7 (57%) tumours exposed *ex vivo* to the agent (Figure. 9B). Furthermore, quantitative immunohistochemistry with antibodies to pRelA^536^ indicated *ex vivo* exposure to low dose aspirin induced NF-κB pathway activation in 3/6 tumours (Figure 7C). Importantly, for individual tumours, there was a very strong inverse correlation (*r*^2^ = –0.85, *n* = 6) between aspirin effects on TIF-IA and RelA^536^ phosphorylation (Figure 9D). That is, the greater the loss of TIF-IA, the greater the increase in NF-κB pathway activation. These data confirm that aspirin causes PolI complex disruption and activates the NF-κB pathway in primary human tumours and suggests a strong relationship between these two events in a whole tissue setting. These data have far reaching implications for understanding of the anti-tumour effects of this agent.

## DISCUSSION

The work presented here has great significance as we identify a novel mechanism by which PolI activity is inhibited by stress, and reveal NF-κB activation as a novel downstream consequence of nucleolar stress response (Figure 9G). We demonstrate the relevance of this pathway *in vivo* using human clinical samples and show it may contribute to the anti-tumour effects of aspirin. These data shed new light on the mechanisms by which nucleoli sense stress and coordinate the downstream consequences.

### Identification of an TIF-IA–NF-κB nucleolar stress response pathway

The paradigm of nucleolar stress response is inhibition of rDNA transcription leading to stabilisation of p53 (6). Here, we provide powerful evidence for an independent nucleolar stress response pathway that is characterised by degradation of TIF-IA leading to activation of NF-κB. Firstly, we demonstrate that specifically disrupting the PolI complex activates the cytoplasmic NF-κB pathway, but that this effect is not mimicked by potent inhibitors of rDNA transcription. Secondly, we show that TIF-IA degradation precedes NF-κB pathway activation in response specific stimuli. Thirdly, using three independent approaches (siRNA depletion of P14ARF, expression of mutant UBF and expression of mutant TIF-IA), we demonstrate that blocking TIF-IA degradation blocks stress effects on the NF-κB pathway. Finally, we demonstrate a strong correlation between loss of TIF-IA and activation of NF-κB in a whole tissue setting. We are currently exploring the mechanisms by which TIF-IA degradation induces NF-κB signalling. Based on the p53 model, we suggest that an NF-κB activating factor(s) is released from nucleoli upon PolI complex disruption. CK2 is an excellent candidate factor as it is found as part of the PolI complex (18) and phosphorylates IκBα in response to UV-C (19). Another kinase of interest is NIK (NF-κB inducing kinase), which acts upstream of the IkappaB kinase (IKK) complex and is known to shuttle through nucleoli (49). The ribosomal proteins L3 and S3 have also been shown to complex with IκB and modulate NF-κB activity (24,49–50). We presume that the p53 regulatory proteins RPL11 and 5 are not involved as they are released from nucleoli in response to CX5461 and BMH21, which we found have no effect on NF-κB signalling.

In support of our notion that the TIF-IA–NF-κB nucleolar stress response pathway is distinct from classical nucleolar stress, we found that it is associated with a distinct nucleolar phenotype. That is, inhibition of rDNA transcription alongside *increased* nucleolar size. This phenotype would appear to contradict the belief, used widely by pathologists, that increased nucleolar size is a marker for enhanced rRNA transcription. In keeping with our findings, Fatyol *et al.* found that MG132 induces a significant increase in nucleolar volume while inhibiting rRNA transcription and mediating cell death (51). Similarly, Bailly *et al* found that the NEDD8 inhibitor, MLN4924, causes an increase in nucleolar size while inducing cell death, although in this case there was no effect on rRNA transcription (52). More recently, Buchwalter *et al* (53) and Tiku *et al.* (54) reported an association between increased nucleolar size and premature aging. This is extremely interesting given the role of NF-κB activity in aging disorders (55). Understanding how TIF-IA degradation alters nucleolar size, and the role this plays in NF-κB signalling, is now a priority.

Since NF-κB stress stimuli inhibit rRNA transcription, it is highly likely that they also stabilize p53. We have previously demonstrated that stimulation of the NF-κB pathway is absolutely necessary for aspirin-mediated apoptosis, but that p53 is dispensable (26,56). Here we show that blocking TIF-IA degradation blocks aspirin-mediated stimulation of the NF-κB pathway and apoptosis. Therefore, we believe that in this context, the phenotypic response to nucleolar stress is governed by the NF-κB pathway.

### Identification of a novel pathway to TIF-IA inactivation

It is well documented that the phosphorylation status of TIF-IA is modulated in response to environmental and cytotoxic stress so that rates of rRNA transcription can be adjusted accordingly (34). Here we identify an alternative mechanism by which stress can act on the PolI complex involving degradation of TIF-IA. We also present several lines of evidence to suggest that this degradation lies downstream of CDK4 inhibition and UBF/p14ARF (Figure 9G). We demonstrate that stress effects on TIF-IA can be mimicked by two highly specific CDK4 inhibitors and by CDK4 depletion. We also demonstrate silencing of UBF or p14ARF blocks aspirin and CDK4i-mediated degradation of TIF-IA, while overexpression of either of these proteins enhances this effect. Furthermore, we show that expression of mutant UBF (S484A) blocks aspirin and CDK4i-mediated TIF-IA degradation. Although these studies were focussed on CDK4, the small molecule inhibitors we used to block CDK4 also block CDK6 activity and so, we cannot rule out a possible role for this kinase.

In addition to CDK4-p14ARF/UBF, we identified a role for serine (S)44 of TIF-IA, and in particular, de-phosphorylation at this site. Mayer *et al* have previously shown that S44 of TIF-IA is dephosphorylated by protein phosphatase 2A (PP2A), and that MTOR inhibition plays a role in this process (37). Here show that the PP2A inhibitor, CalyculinA, blocks aspirin and CDK4i-mediated TIF-IA degradation, consistent with a role for this phosphatase. However, we found that the specific MTOR inhibitor, rapamycin, has a minimal effect on basal TIF-IA levels and no effect on aspirin-mediated degradation of the protein (Supplemental Figure S5C). These data would suggest that an alternative, PP2A dependent pathway may be involved. Defining the nature of this pathway, and how it combines with CDK4 inhibition-UBF/p14ARF to promote TIF-IA degradation, is out-with the scope of the current study. However, future understanding of this pathway is critical as it may reveal novel targets to modulate TIF-IA levels and hence, NF-κB activity and rRNA transcription.

### Aspirin degradation of TIF-IA:Identification of a novel mechanism of action

Despite the burden of evidence indicating aspirin use can prevent colorectal cancer, the agent cannot be recommended for this purpose due to its significant side effect profile. Hence, efforts are now focussed on understanding the mechanism by which aspirin acts against colorectal cancer cells to identify markers of response and targets for safer, more effective alternatives. Uncontrolled rDNA transcription is a hallmark of colorectal and other cancers and contributes to tumour growth by allowing de-regulated protein synthesis and uncontrolled activity of nucleolar cell growth/death pathways (33,57). Here we show for the first time that aspirin inhibits rDNA transcription in colorectal cancer. We show this in multiple colorectal cancer cell lines and demonstrate the agent induces TIF-IA degradation in a whole tumour setting. These data represent an extremely exciting, novel mechanism of action that could have a significant impact in defining patients that would benefit from aspirin therapy.

We show aspirin activates the NF-κB pathway both *in vitro*, and in a whole tissue setting. While this agent is generally thought to be an inhibitor of NF-κB activity, its effects are context dependent. That is, short pre-exposure inhibits cytokine-mediated activation of NF-κB while prolonged exposure to the agent alone (representing chemoprevention) activates NF-κB signalling (58,26). These data would suggest that the pathway by which NF-κB is activated in response to nucleolar stress differs from that utilised by inflammatory cytokines, although this remains to be confirmed.

In summary, the data presented here open up new avenues of research into nucleolar regulation of NF-κB signalling and regulation of TIF-IA stability under stress. They also shed further light on the complex mode of action of aspirin and related non-steroidal anti-inflammatory drugs (NSAIDs).

## Supplementary Material

Supplementary Data
