# Supplementary material for: Identification of a novel TIF-IA–NF-κB nucleolar stress response pathway
Source: Nucleic Acids Res. 2018 Jun 5;46(12):6188–205. doi: 10.1093/nar/gky455 (PMC6158704; doi:10.1093/nar/gky455)
Supplement: Supplementary Data [file gky455_supplemental_files.pdf]

Supporting Figure Legends

Supplemental Figure 1: Disruption of the PolI complex stimulates NF-κB signalling in a manner dependent upon IκB degradation. (A and C) HCT116 cells were transfected with the indicated siRNA species **(A)** Cells were co-transfected with pCMVβ, 3x κB ConA-Luc or an equivalent plasmid with κB sites deleted (ConAΔκB). Graph depicts % relative luciferase activity compared to cells transfected with 3x κB ConA-Luc and siControl. The mean of three individual repeats (± s.e.m) is shown. **(C)** qRT-PCR with primers for the 47S pre-rRNA transcript measured levels of rDNA transcription. GAPDH was used as a normalising gene. Results are presented as the percentage of relative 47S transcription compared to control siRNA. The mean (± s.e.m) is shown. N=3. **(B)** SW480 cells were transfected with the indicated siRNA species. qRT-PCR was performed with primers for the NF-κB target gene IκBα. GAPDH was used to normalise. Results are presented as the fold increase in transcript compared to siControl. The mean (± s.e.m) is shown. N= 2 biological repeats. **(D)** Parental HRT18 cells (IκB WT), and cells expressing mutant IκB resistant to stimuli-induced degradation (IκB-SR), were transfected with the indicated siRNA species, pCMVβ and 3x κB ConA-Luc. Relative luciferase activity was determined and presented as in (A). Mean ± s.e.m is shown (N=3). Inset: Immunoblot shows equally efficient depletion of TIF-IA and UBF in both cell lines. Actin acts as a control. *P* values throughout are compared to the respective control and were derived using a two tailed students T test. See also Figure 1.

Supplemental Figure 2: TIF-IA degradation and induction of an atypical nucleolar phenotype in response to aspirin. (A and B) Pancreatic (PNT), colorectal adenocarcinoma (RKO, HCT116) and the equivalent in which the p53 gene has been deleted by homologous recombination ($HCT116^{p53-/-}$), were treated with aspirin (10mM for the times specified, or at the concentrations specified for 16h). Western blot analysis was performed with the indicated antibodies on whole cell lysates (WCLs). **(C)** Immunoblot analysis was performed on cytoplasmic (IκB) or WCLs (TIF-IA) from aspirin (10mM) treated cells, as described in Figures 2C and D. ImageJ analysis quantified band intensities relative to the respective control. Graph represents the mean of 3 IκB experiments and 4 TIF-IA experiments (+/-s.e.m). **(D)** qRT-PCR measured tif-ia gene transcription in response to aspirin (10mM for the times specified). Results are presented as the percentage of relative TIF-IA mRNA compared to 0h control. The mean (± s.e.m) is shown. N=3. **(E to H)** Aspirin-mediated TIF-IA degradation is independent of MDM2 and dependent on lysosomal and proteasomal pathways. SW480 cells were pre-treated for 2h with DMSO (carrier), the MDM2 inhibitor, Nutlin3 (5μM), MG132 (25μM), Lactacystin (35 μM), Quinacrine (25μM), BafilomycinA1 (100nM) or MG132 (10μM) plus quinacrine (25μM). Following aspirin treatment (10mM, 4h or as indicated), TIF-IA levels were monitored by Western blot analysis. **(I)** SW480 cells were transfected with GFP-TIF-IA then treated with aspirin (10mM) for the times specified. Immunoblot was performed with α GFP antibody. Actin acts as a loading control throughout. *P* values are compared to the respective control and were derived using a two tailed students T test.

Supplemental Figure 3: Aspirin modulates the structure and function of the nucleolus. (A) SW480 cells were subjected to fluouridine (FUrd) run on after treatment with carrier (NT), aspirin (10mM, 8h) or actinomycinD (ActD. 2h, 50ng/Ml). Images were captured (top) and analysed for FUrd incorporation using ScanR image analysis software (botom). The results are presented as the percentage incorporation compared to basal (NT) levels. The mean (± s.e.m) of at least 1000 cells in three independent experiments is shown. **(B)** Immunomicrograph demonstrating relocalisation of nucleolar proteins fibrillarin and POLR1A in fixed cells following exposure to aspirin (10mM, 8h). Actin acts as a loading control throughout. *P* values

are compared to the respective control and were derived using a two tailed students T test. Scale bars =10µm. See also Figure 2.

Supplemental Figure 4: Inhibition of CDK4 activity induces TIF-IA degradation (A-D) Anti-TIF-IA immunoblots performed on whole cell lysates from SW480 cells: **(A)** Treated with the specific CDK4 inhibitor, PD-0332991 (2uM), for the times specified. **(B)** Transfected with the indicated siRNA species. **(C and D)** Pre-treated with MG132, Quinacrine or Nutlin-3, as above, prior to exposure to the small molecule CDK4 inhibitor, 2-bromo-12,13-dihy-dro-indolo[2,3-a]pyrrolo[3,4-c] carbazole-5,7(6H)-dione (CDK4i, 2uM) for the times specified. **(E)** Treated with CDK4i (2uM) or Roscovitine (50uM) for 24h. Actin acts as a loading control throughout. **(F)** Representative DNA histograms demonstrating the cell cycle status of SW480 cells treated as indicated for 48h (N=3).

Supplemental Figure 5: A role for p14ARF in TIF-IA degradation. (A) SW480 cells were treated with 10mM aspirin for the times specified. Immunoprecipitation was carried out on WCL using antibodies to TIF-IA. Precipitated proteins were subjected to western blot analysis with antibodies to p14ARF and TIF-IA. Input levels of the indicated proteins are shown. **(B)** Degradation of TIF-IA precedes loss of p14ARF in response to aspirin. SW480 cells were treated with aspirin (10mM) for the times specified. Western blot analysis was performed with the indicated antibodies. Actin acts as a loading control. **(C)** SW480 cells were pre-treated for 2h with Rapamycin as indicated then treated with aspirin (10mM) for 0 or 4h. Immunoblot was performed on WCE with the indicated antibodies. pS6K acts as a control for mTOR inhibition by rapamycin.

Supplemental Figure 1

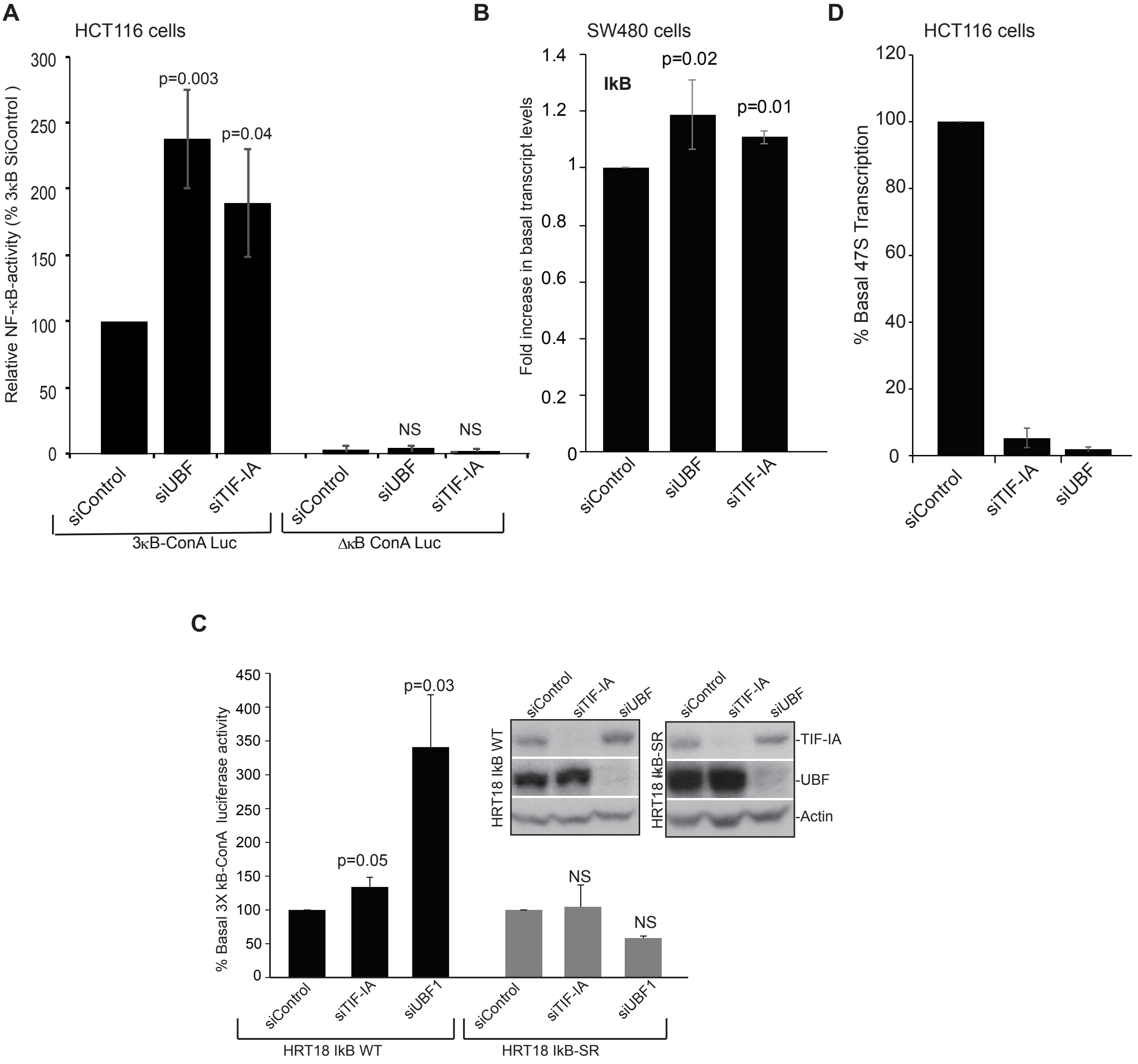

Supplemental Figure 2

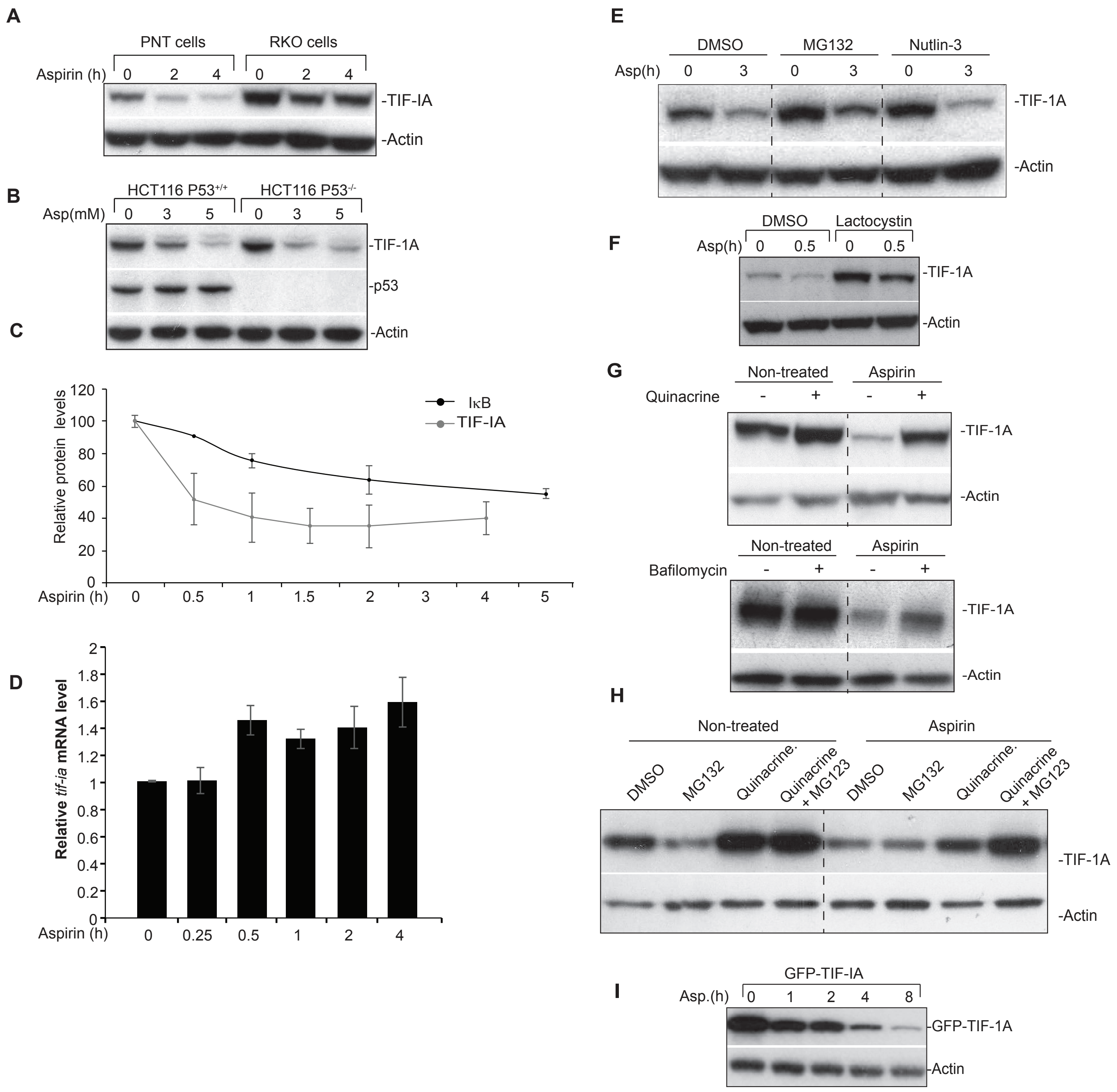

Supplemental Figure 3

A

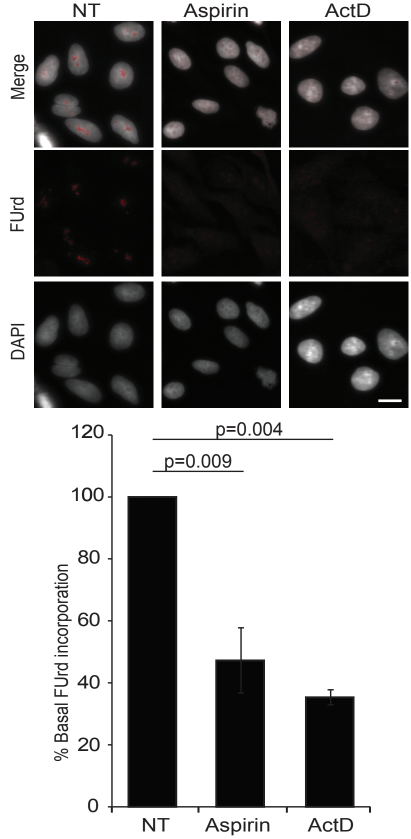

B

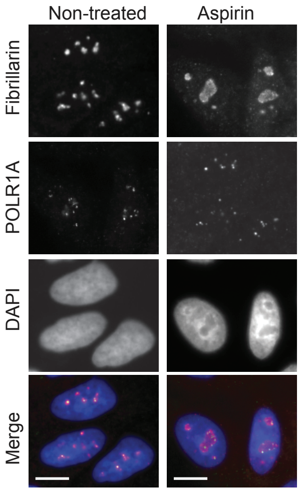

Supplemental Figure 4

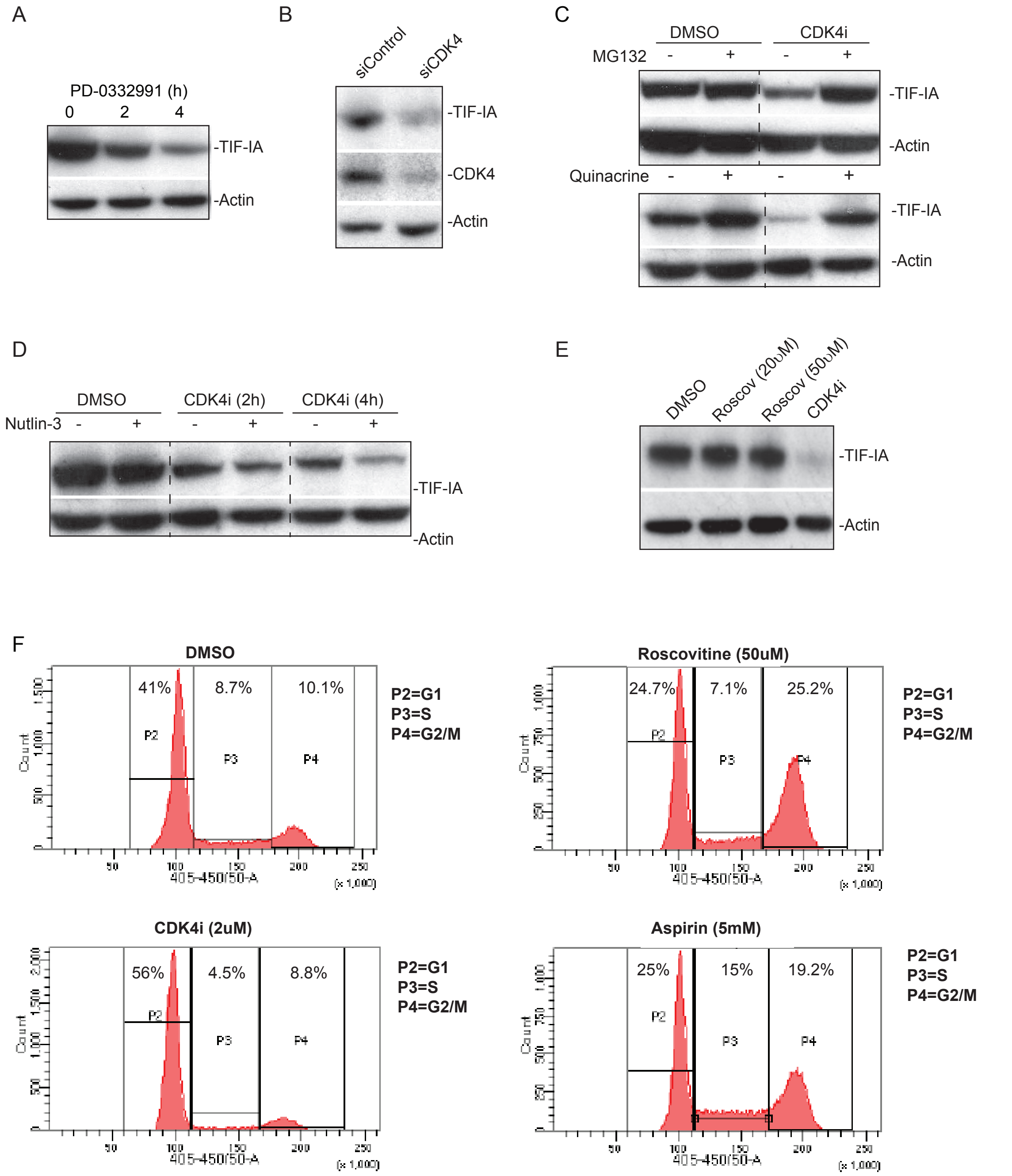

Supplemental Figure 5

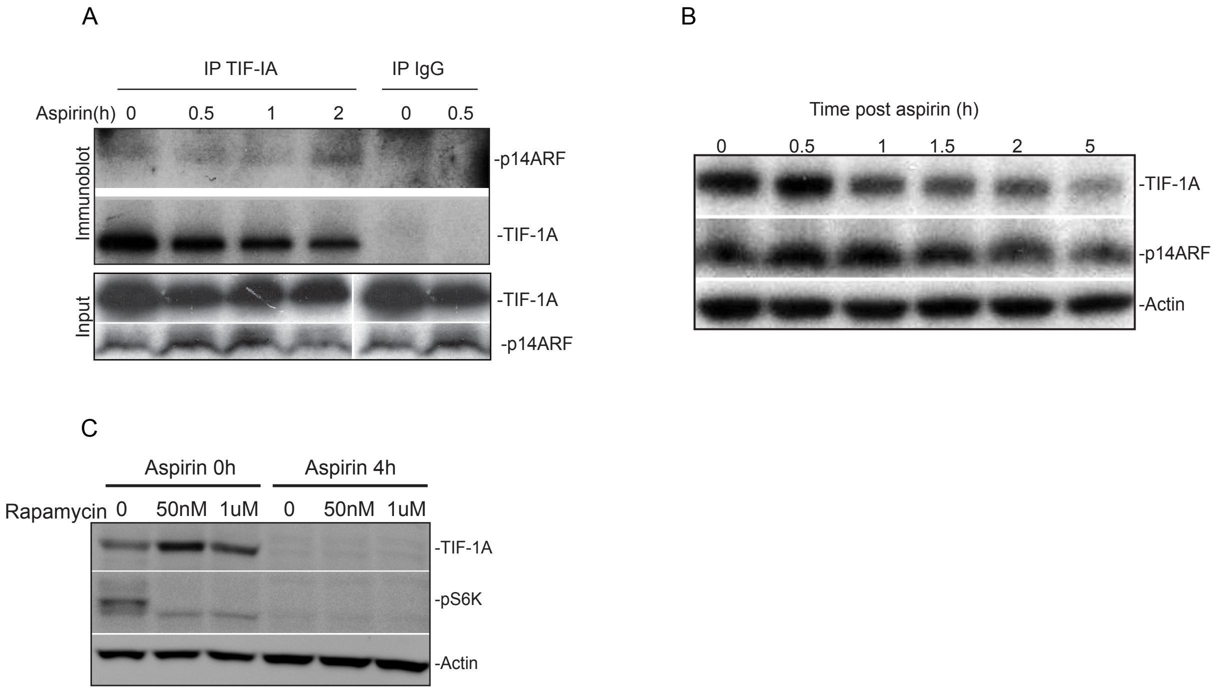

Stress type	Stimulus	Activation NF-kB	Reference	Nucleolar disruption	Reference
DNA damage/ genotoxic stress	UV-C	y	Wu and Miyamoto, 2007;Li et al, 1998;	y	Al-Baker et al, 2004; Moore et al, 2011, Lamond, 2010; Kruklak et al, 2007; Rubbi and Milner , 2003
	IR (DSB)	y		y	
	Camptothecin	y	Piret et al, 1996, Wu and Miyamoto,	y	
	Bleomycin	y		y	
Oxidadtive stress	ROI	y	Schreck et al, 2001	y	Wang et al, 2012; Mekhail et al, 2003; Rubbie and Milner, 2003
	Hypoxia	y	Kenneth and Rocha, 2008	y	
Chemo-therapeutic /-preventative agents	Daunorubicin/ Doxorubicin	y	Bian et al, 2001;Campbell et al, 2006	y	Burger et al, 2010; Chan et al, 2007
	Etoposide	y	Tabata et al, 2001; Piret et al, 1996	y	Boisvert et al, 2010
	Aspirin/ NSAIDs	y	Stark et al, 2001; Loveridge et al, 2008	y	Stark and Dunlop, 2005; Loveridge et al, 2008
Nutrient stress	Serum starvation	y	Mogi et al, 2004;Stark and Dunlop, 2005	y	Mayer and Grummt, 2006
Oncogenic stress	Ras/BCR-ABL	y	Basseres and Baldwin, 2006; Perkins and Gilmore, 2006	y	Rugerro, 2012; Suzuki et al, 2012
